# Manipulation of intestinal epithelial cell function by the cell contact-dependent type III secretion systems of *Vibrio parahaemolyticus*

**DOI:** 10.3389/fcimb.2013.00114

**Published:** 2014-01-10

**Authors:** Nicky O'Boyle, Aoife Boyd

**Affiliations:** Pathogenic Mechanisms Research Group, Microbiology, School of Natural Sciences, National University of Ireland GalwayGalway, Ireland

**Keywords:** TTSS, MshA, *Vibrio parahaemolyticus*, effector, intestinal cells

## Abstract

*Vibrio parahaemolyticus* elicits gastroenteritis by deploying Type III Secretion Systems (TTSS) to deliver effector proteins into epithelial cells of the human intestinal tract. The bacteria must adhere to the human cells to allow colonization and operation of the TTSS translocation apparatus bridging the bacterium and the host cell. This article first reviews recent advances in identifying the molecules responsible for intercellular adherence. *V. parahaemolyticus* possesses two TTSS, each of which delivers an exclusive set of effectors and mediates unique effects on the host cell. TTSS effectors primarily target and alter the activation status of host cell signaling proteins, thereby bringing about changes in the regulation of cellular behavior. TTSS1 is responsible for the cytotoxicity of *V. parahaemolyticus*, while TTSS2 is necessary for the enterotoxicity of the pathogen. Recent publications have elucidated the function of several TTSS effectors and their importance in the virulence of the bacterium. This review will explore the ability of the TTSS to manipulate activities of human intestinal cells and how this modification of cell function favors bacterial colonization and persistence of *V. parahaemolyticus* in the host.

## Introduction

*Vibrio parahaemolyticus* is the world's leading causes of seafood-borne gastroenteritis. The organism is found in marine and estuarine environments and can be found in the water column or associated with sediments, shellfish, and zooplankton (Su and Liu, 2007). In immuno-competent individuals the clinical manifestations of *V. parahaemolyticus* infection are mild, self-limiting gastroenteritis accompanied by abdominal cramps and watery diarrhea which can last for 2–3 days (Daniels and Shafaie, 2000; Nair et al., 2007; Honda et al., 2008). In some cases however, severe inflammatory diarrhea occurs, with major destruction of gastric and intestinal epithelia, and blood being shed in the stools (Honda et al., 2008). Such cases can occur due to underlying health conditions and may result in septicaemia and subsequent death of infected individuals. The most common route of infection is through the consumption of raw or undercooked seafood. The majority (88%) of reported cases of *V. parahaemolyticus* infection in the United States between 1988 and 1997 were due to the consumption of raw oysters (Daniels et al., 2000). *V. parahaemolyticus* has also been reported to cause wound infections which can lead to necrotizing fasciitis, a severe skin infection which results in extensive tissue damage (Ralph and Currie, 2007; Tena et al., 2010).

Upon entry into the human body *V. parahaemolyticus* will encounter enterocytes lining the intestinal tract. These are the primary attachment sites of the bacteria. Also within the intestinal epithelium are M-cells with a high capacity for bacterial transcytosis and through which *V. parahaemolyticus* can travel from the lumen to the underlying tissues (Finn et al., 2013). Below the epithelial layer is the lamina propria—a layer of connective tissue through which passes small blood vessels, lymphatic ducts and nerve fibers (Madara, 2010). The intimate intercellular interactions between proteins on adjoining enterocytes constitute tight junctions and contribute to the physical barrier function of the intestinal epithelium (Assimakopoulos et al., 2011). The gastrointestinal epithelium also functions as a selectively permeable barrier controlling the passage of nutrients, water and ions. The microvilli on the apical surface of the enterocytes greatly increase the cell surface area, allowing efficient absorption and transport of small molecules (Pastorelli et al., 2013). By modulating and/or disrupting intestinal epithelial cell function, gastrointestinal pathogens can increase non-selective intestinal permeability, modulate ion and fluid efflux and allow bacterial access to deeper tissues and interfere with host immune responses.

The genome of the pandemic clone of *V. parahaemolyticus* was sequenced in 2003 (Makino et al., 2003) and since then research has targetted efforts to unravel the mechanisms by which this pathogen exerts its inflammatory diarrheagenic effects on intestinal tissues. The thermostable direct hemolysin (TDH) had long been considered a marker of virulence due to its detection in the majority of clinical isolates (Miyamoto et al., 1969). However, a number of hemolysin negative strains were isolated from patients suffering from gastroenteritis, indicating the involvement of alternative virulence factors in *V. parahaemolyticus* pathogenicity (Nishibuchi et al., 1992). Perhaps the most striking observation from the sequencing of the *V. parahaemolyticus* genome was the detection of two Type Three Secretion Systems (TTSS), one located on each chromosome, a finding which indicated a molecular mechanism of virulence distinct from that of *Vibrio cholerae* and shifted focus from predominantly TDH-centerd research to molecular characterization of *V. parahaemolyticus* TTSS (Makino et al., 2003). The identification of the TTSS finally offered a clue as to the alternative virulence factors which were predicted to play a role in TDH-independent cytotoxicity and enterotoxicity.

Bacterial TTSS have been referred to as nanomachines, capable of efficiently delivering effector proteins into host cells in order to hijack host cell signaling, thereby manipulating a variety of host cell functions (Cornelis, 2006). *V. parahaemolyticus* TTSS1 belongs to the Ysc family of TTSS and is ancient in origin, while TTSS2 resides in a pathogenicity island which also contains the *tdhA* and *tdhS* alleles and belongs to the Hrp1 family (Makino et al., 2003; Troisfontaines and Cornelis, 2005). The TTSS machinery comprises a secretion apparatus traversing the bacterial membranes, a translocation pore to traverse the eukaryotic membranes and a needle to connect the two parts. While the TTSS machinery is highly conserved within each family, the effector proteins responsible for modulating host cell responses are variable, thereby yielding widely diverse functionality even within each family (Troisfontaines and Cornelis, 2005). The TTSS effectors of *V. parahaemolyticus* are potent molecules enabling the bacterium to colonize its host. TTSS2 effectors impair epithelial cell function and structure resulting in damage to the intestinal lining, inflammation and diarrhea. TTSS1 effectors are important for systemic *V. parahaemolyticus* infections and may determine the final outcome of disease. In order for these effectors to be channeled into the host cell, the bacterium must engage in a tight association with the eukaryotic cell surface. This cell-contact is mediated by the binding of bacterial adhesins to host receptors, thereby stimulating formation of the TTSS translocation pore in the eukaryotic membrane and operation of a fully functional TTSS machinery.

This review will describe the processes which are involved in the pathogenesis of *V. parahaemolyticus* in intestinal epithelial tissues, with specific reference to the adhesins mediating adherence to these tissues and the roles of the TTSS effector proteins which manipulate cell function during intestinal infection.

## Virulence factors involved in intestinal colonization by *V. parahaemolyticus*

A number of recent studies have aimed to identify the virulence factors involved in the attachment and colonization of intestinal tissues by *V. parahaemolyticus*. As efficient binding to the host cell is a pre-requisite for TTSS functionality and pathogenesis, this critical phase of infection offers a promising target for medical intervention. Bacterial adherence to host tissues is a complex process involving the synergistic actions of multiple bacterial adhesins with host cell receptors, forming an intimate multivalent connection between pathogen and host. The gastro-intestinal tract is a highly dynamic environment, with varying mucus density, commensal flora composition and a wide diversity of cell types. For this reason, successful pathogens such as *V. parahaemolyticus* have evolved multiple adhesion mechanisms to allow for flexible and efficient attachment throughout the intestine. Deciphering the diverse molecular interactions governing this critical phase of pathogenesis is critical for the development of strategies to combat *V. parahaemolyticus* infection.

In order to fully appreciate the significance of these adhesins in the overall pathogenicity of *V. parahaemolyticus*, it is important to consider how disruption of adherence can interfere with pathogenesis in infected cells. The mannose sensitive hemagglutinin (MSHA) Type IV Pilus and multivalent adhesion molecule 7 (MAM7) are important for attachment of *V. parahaemolyticus* to eukaryotic intestinal cells *in vitro* and consequently for downstream TTSS-mediated pathogenic effects on host cells (Krachler et al., 2011; O'Boyle et al., 2013). The observation of an integral link between adhesin function and downstream TTSS-dependent pathogenicity reflects the key role of cell contact in the pathogenesis of *V. parahaemolyticus*.

### TYPE IV pili (TFP)

TFP are long (>1 μm), narrow (5–8 nm), filamentous structures which extend outward from the bacterial cell and are composed of thousands of major and minor pilin subunits (Craig et al., 2004). TFP from numerous pathogenic bacteria have been demonstrated as playing integral roles in attachment to epithelial tissues (Attridge et al., 1996; Cleary et al., 2004; Hélaine et al., 2005; Giltner et al., 2006). The sequencing of the *V. parahaemolyticus* genome revealed distinct operons encoding two different type IVa pili (Makino et al., 2003). The genes for the chitin-regulated pilus (ChiRP)—whose major pilin is PilA—were scattered throughout chromosome 1, as is typical for type IVa pili (Aagesen and Hase, 2012). The MSHA pilus by comparison was encoded within a single discrete genetic locus, a feature which is more commonly associated with type IVb pili (Aagesen and Hase, 2012). As the name suggests, chitin is required for induction of PilA transcription and ChiRP production, while the MSHA pili are produced under normal laboratory growth conditions (Shime-Hattori et al., 2006; O'Boyle et al., 2013). These pili are termed MSHA pili by virtue of similarity with the pilus encoding operon of *V. cholerae*. It has been suggested however, that the nomenclature for the *V. parahaemolyticus* pilus is inappropriate, as unlike the true MSHA possessed by *V. cholerae*, the pili from *V. parahaemolyticus* do not bind to mannose or agglutinate red blood cells (Nakasone and Iwanaga, 1990; O'Boyle et al., 2013).

The ChiRP and the MSHA pilus of both *V. cholerae* and *V. parahaemolyticus* have been implicated in biofilm formation (Watnick et al., 1999; Shime-Hattori et al., 2006). Although the *V. cholerae* MSHA pilus was demonstrated as playing no role in intestinal colonization in either murine or human infections (Attridge et al., 1996; Thelin and Taylor, 1996; Tacket et al., 1998), differential major pilin sequence composition coupled with differential hemagglutination functionality led to further investigation of the *V. parahaemolyticus* MSHA pilus as a host cell binding adhesin.

**Figure 1:**
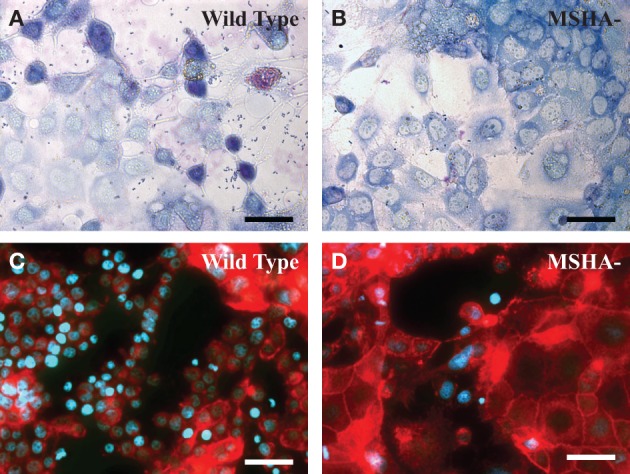
**TTSS-associated morphological alterations in intestinal cells require a functional MSHA pilus. (A,B)** Giemsa stained co-cutures of wild type **(A)** or Δ*mshAl* **(B)** *V. parahaemolyticus* and Caco-2 following 1 h of incubation and removel of non-adherent bacteria. **(C,D)** Phalloidin- Alexa 568/Hoechst 33342 stained Caco-2 visualized by epifluorescence microscopy following 2.5 h of infection with wild-type **(C)** or Δ*mshAl* **(D)** *V. parahaemolyticus.* Scale bars represent 50 μm.

MSHA deficient mutants of *V. parahaemolyticus* adhered at significantly lower efficiency to differentiated intestinal Caco-2 cells than the wild type (Figures 1A,B), (O'Boyle et al., 2013). This finding is supported by early observations that purified MSHA pili of *V. parahaemolyticus* adhered with high efficiency to intestinal explants from rabbits (Nakasone and Iwanaga, 1990). Although at the time of that study, the pilus encoding genes and pilus protein composition were not known, the pili were found to be immunogenic and the binding of *V. parahaemolyticus* to rabbit intestinal tissue could be inhibited by pre-treatment with anti-pilus antibodies (Nakasone and Iwanaga, 1990, 1991; Nakasone et al., 2000). The lower rate of bacterial adherence corresponded with decreased levels of cellular uptake indicating that adherence to host cells is an important pre-requisite for cellular invasion (O'Boyle et al., 2013). Following confirmation of adhesin functionality of MSHA, potential receptors were identified using a glycan microarray screening technique. Mutants that were devoid of MSHA pili were defective in binding to a group of structurally similar glycans including: Lewis A and X, and blood groups A and B (O'Boyle et al., 2013). As these glycans are expressed in intestinal tissues (Mollicone et al., 1985), they may serve as intestinal receptors for the MSHA pilus (O'Boyle et al., 2013).

A 20% decrease in *V. parahaemolyticus*–mediated cytotoxicity was observed with deletion of MSHA pilus components (O'Boyle et al., 2013). A number of other host cell markers of *V. parahaemolyticus* pathogenicity were also investigated, with delayed cell rounding, reduced nuclear condensation and decreased IL-8 secretion all being associated with disruption of the MSHA pilus (O'Boyle et al., 2013). The decreased cell rounding effect of MSHA deficient *V. parahaemolyticus*—which likely occurs due to diminished secretion of the TTSS1 effector VopS—is clearly evident in Figures 1C,D. Each of these pathogenic effects has been attributed—at least in part—to TTSS effectors. As such, interference with initial attachment to the host cell provides an efficient means of disturbing the injection of effectors during infection.

### Multivalent adhesion molecule 7 (MAM7)

Like the MSHA pilus, the MAM7 adhesin facilitates initial attachment with the host cell (Krachler et al., 2011). This protein is present in the outer-membrane of *V. parahaemolyticus* and possesses seven mammalian cell entry (Mce) domains (Krachler et al., 2011). Deletion of MAM7 resulted in a 60% decrease in adherence of bacteria to intestinal Caco-2 cells and caused similar decreases in attachment to HeLa, RAW 264.7 macrophages and 3T3 fibroblasts (Krachler et al., 2011). Host cell attachment was mediated via interaction between the Mce domains of MAM7 and two extracellular matrix components—the protein fibronectin and the phospholipid phosphatidic acid (Krachler et al., 2011). At least five Mce domains were required for binding to fibronectin, while basic amino acids within the Mce domains were required for binding to phosphatidic acid (Krachler and Orth, 2011). Binding to fibronectin and phosphatidic acid was not mutually exclusive and binding likely resulted in the formation of a tripartite complex (Krachler and Orth, 2011). The decrease in adherence upon deletion of *mam7* in *V. parahaemolyticus* correlated with a 20% decrease in the bacterium's cytotoxicity (Krachler et al., 2011). In a complementary toxicity assay, upon feeding of *Caenorhabditis elegans* nematodes with wild type bacteria, rapid lethality, severe growth retardation and intestinal rupture occurred, while mutants lacking MAM7 did not affect life expectancy, growth rate or intestinal integrity of the nematodes (Krachler et al., 2011).

*V. parahaemolyticus* MAM7 and homologues from the enteric pathogens *V. cholerae*, *Yersinia pseudotuberculosis* and enteropathogenic *E. coli* could be expressed functionally in *E. coli* BL21, such that expression resulted in increased adherence of the organism to Caco-2 (Krachler et al., 2011). This indicated that expression of MAM7 by non-pathogenic bacteria could potentially be used as a prophylactic to prevent the attachment of pathogenic microorganisms. Indeed, the authors observed significant decreases in cytotoxicity due to infection with each of these enteric pathogens following pre-treatment of epithelial monolayers with *E. coli* BL21 expressing MAM7 (Krachler et al., 2011). This potential for prophylactic use of MAM7 was further developed by the observation that pre-treatment of epithelial cells with bead immobilized MAM7 could impede the binding of the opportunistic pathogen *Pseudomonas aeruginosa* (Krachler et al., 2012). The work carried out by this group provides an excellent example of the exploitation of a *V. parahaemolyticus* virulence factor for the broad range prevention of pathogen binding.

While a definitive link between adhesion and pathogenicity has been established for MAM7 and the MSHA pilus, a number of other proteins such as the cell-associated hemagglutinin (cHA) type VI secretion systems (T6SS), capsular polysaccharides (CPS) and GlcNAc binding protein (GbpA) have been confirmed as having adhesin functionality on intestinal cells *in vitro* (Nagayama et al., 1995; Hsieh et al., 2003; Kirn et al., 2005; Yu et al., 2012), and as such warrant further investigation as mediators of cell contact-dependent pathogenicity.

### Cell-associated hemagglutinin (cHA)

As previously mentioned, the MSHA pilus of *V. parahaemolyticus* does not have mannose-binding or hemagglutinin functionality (Nakasone and Iwanaga, 1990; O'Boyle et al., 2013). A variety of hemagglutination positive *V. parahaemolyticus* strains however, have been isolated, and in these cases, hemagglutination was facilitated by a cell-associated non-pilus hemagglutinin (Nagayama et al., 1994). Nagayama et al. (1994) found a direct correlation between the ability of a variety of *V. parahaemolyticus* strains to agglutinate red blood cells in a mannose-sensitive manner and the ability to bind to Caco-2 intestinal cells. Interestingly, no correlation between adhesiveness and piliation was observed. A 26 kDa protein responsible for hemagglutination was purified (Nagayama et al., 1995). It was shown by immunogold labeling to be located on the cell surface, and as such was deemed a cell-associated hemagglutinin (cHA) (Nagayama et al., 1995). Adherence to cultured rabbit enterocytes was efficiently inhibited by antibodies raised against purified cHA and by d-mannose, indicating that cHA was responsible for both mannose sensitive hemagglutination and adherence to rabbit enterocytes. For this reason Nagayama et al. (1995) stated “Thus, we do not believe that pili of *V. parahaemolyticus* participate in the adherence.” This hypothesis clearly contradicts the findings of Nakasone and Iwanaga (1990) and O'Boyle et al. (2013) who observed that pili were indeed mediators of *V. parahaemolyticus* adherence to rabbit and human intestinal tissues and therefore indicates that adherence mechanisms of *V. parahaemolyticus* are likely strain specific. cHA function has not been described in sequenced strains of *V. parahaemolyticus*, nor has the identification of the gene encoding cHA been reported. As such it remains unclear whether or not cHA plays a role in adherence-mediated virulence of pandemic *V. parahaemolyticus* strains.

### Type VI secretion systems (T6SS)

The most recently identified Gram-negative bacterial secretion system is the T6SS (Pukatzki et al., 2006). These systems consist of a multi-component “cell-puncturing needle” and function in a similar manner to the TTSS by allowing the injection of effector proteins from the bacterial cell into a target cell (Coulthurst, 2013). Two distinct *V. parahaemolyticus* T6SS were recently identified, with T6SS1 being found predominantly in clinical isolates and T6SS2 being found in both clinical and environmental isolates (Yu et al., 2012). T6SS1 has been characterized as an anti-microbial secretion system—targeting prokaryotic as well as eukaryotic cells, as a means of out-competing other microbial species, thereby improving environmental fitness (Salomon et al., 2013). T6SS1 also has a role in adherence to eukaryotic cells with deletion of IcmF, the major secretion tube protein or Hcp1, a T6SS tip located translocator, leading to a 50% decrease in adherence to Caco-2 (Yu et al., 2012). The T6SS of *Campylobacter jejuni* has a similar role in intestinal colonization, with deletion mutants showing reduced rates of adherence and invasion in T84 human colonic cells and decreased rates of intestinal colonization in infected mice (Lertpiriyapong et al., 2012). The mechanism by which these secretion systems regulate colonization of intestinal cells has not been established, however, it is tempting to speculate that a similar system to the enteropathogenic *E. coli* (EPEC) Tir-intimin regulated adherence may be at play. The Tir effector protein is secreted into host cells by EPEC, where it then inserts into the apical membrane to act as a receptor for the bacterial adhesin intimin (Kenny et al., 1997). Many bacterial effectors become inserted in host cell membranes following translocation (Dean and Kenny, 2009; Matsumoto and Young, 2009; Mcghie et al., 2009; Figueira and Holden, 2012), as such it is possible that a T6SS effector may enhance adherence by operating as a membrane embedded receptor for a bacterial adhesin, although this has not been shown experimentally.

### GlcNAc binding protein a (GbpA)

Chitin is an oligosaccharide compound consisting of repeating GlcNAc subunits and is a major constituent of the exoskeletons of crustaceans, molluscs and zooplankton—organisms which are commonly colonized by *V. parahaemolyticus*. Chitin and monomeric GlcNAc are recognized as potent activators of transcription in the *Vibrionaceae* (Meibom et al., 2004; Thompson et al., 2011). One gene identified as being activated in the presence of chitin was the *V. cholerae* GlcNAc binding protein encoding *gbpA*. Deletion of GbpA_VC_ decreased attachment of bacteria to crustacean exoskeletons, bead immobilized chitin and importantly cultured HT-29 mucus-producing colonic adenocarcinoma cells (Kirn et al., 2005). This finding linked attachment for the purpose of environmental persistence and gastro-intestinal adherence. Further characterization of GbpA_VC_ revealed that the intestinal mucins MUC2, MUC3 and MUC5AC served as functional receptors for GbpA_VC_ (Bhowmick et al., 2008). Interestingly, purified mucins were capable of inducing transcription of *gbpA* and binding of GbpA_VC_ to HT-29 cells increased mucus production, thereby allowing a cooperative means of enhancing bacterial attachment (Bhowmick et al., 2008).

While little research has been carried out investigating the involvement of *V. parahaemolyticus* GbpA_VP_ in the colonization of intestinal cells, GbpA_VP_ and GbpA_VC_ share 70% amino acid identity and as such conserved functionality is a possibility. GbpA_VP_ is activated by surface sensing, following impedance of polar flagellum rotation (Gode-Potratz et al., 2011). Interestingly surface sensing also activated the transcription of TTSS1 genes, strengthening the hypothesis that GbpA_VP_ may act as a virulence factor in *V. parahaemolyticus* (Gode-Potratz et al., 2011). Surface sensing caused repression of some MSHA genes indicating that MSHA may be important only for the early stages of attachment, with induction of other adhesins occurring later (Gode-Potratz et al., 2011). Further research into GbpA_VP_'s binding specificity and role in pathogenesis is required in order to confirm its functionality as a virulence factor.

### Capsular polysaccharide (CPS)

*Vibrio parahaemolyticus* displays phase variation, modulating between opaque colonies which produce large amounts of CPS and translucent colonies which produce little CPS (Enos-Berlage et al., 2005). Parallel infections of Int-407 intestinal cells identified that opaque colony variants were ten-fold more adhesive than translucent variants (Hsieh et al., 2003). Purified CPS was found to adhere to Int-407 cells and antibodies against purified CPS prevented bacterial adhesion in a dose-dependent manner (Hsieh et al., 2003). A single K8 CPS serotype was investigated in that study. The link between CPS production and intestinal colonization remains tentative as few studies have been carried out in this area and due to CPS diversity detailed analysis of multiple serotypes is warranted.

### Host cell contact in the context of overall pathogenesis

*In vitro* studies have described integral roles for the MSHA pilus and MAM7 in pathogenicity (Krachler et al., 2011; O'Boyle et al., 2013). MAM7 and the MSHA pilus have both been shown to be constitutively expressed prior to attachment “allowing the bacteria to be primed for immediate attachment when encountering a host cell” (Nakasone and Iwanaga, 1990; Krachler et al., 2011; O'Boyle et al., 2013). Other adhesins, such as GbpA may be induced following docking on the cell surface, enhancing the intimacy of binding to the intestinal epithelium. Inhibition of multiple adhesins in the same background would yield interesting insight into the combinatorial effect of the many adhesins possessed by *V. parahaemolyticus* during interaction with intestinal tissues. *In vivo* analysis of the functionality of these adhesins will improve our understanding of the mechanisms by which *V. parahaemolyticus* specifically targets intestinal tissues. While the study of *V. parahaemolyticus* adhesins yields interesting insights into colonization, downstream subversion of host cell function via TTSS activity is responsible for the tissue disruption and inflammatory diarrhea associated with *V. parahaemolyticus* infection. The following sections will describe the functionality of the two TTSS of *V. parahaemolyticus* together with a detailed description of the effector proteins which have been characterized to date.

## *V. parahaemolyticus* effects on the intestinal epithelium and role of the TTSS

*V. parahaemolyticus* disruption of the intestinal epithelium during infection is evident in human biopsies and is a key feature of *V. parahaemolyticus* pathogenesis (Qadri et al., 2003). Intestinal epithelium disruption also occurs in infected ligated ileal loops from adult rabbits and in orally infected infant rabbits (Park et al., 2004b; Ritchie et al., 2012). Both the epithelial damage and the inflammatory response upon *V. parahaemolyticus* infection are more severe than that seen in patients with cholera (Nelson et al., 1976). Evidence suggests that disruption arises as a result of the host inflammatory response to infection in combination with specific *V. parahaemolyticus* virulence mechanisms, such as the TTSS.

Evidence of inflammation during disease in humans is illustrated by increased levels of the pro-inflammatory cytokines Tumor Necrosis Factor-α (TNF-α) and Interleukin-1β (IL-1β) in the stool of *V. parahaemolyticus*–infected patients and by histological visualization of inflammation in intestinal biopsies collected during the acute stage of infection when symptoms, such as diarrhea, are most evident (Qadri et al., 2003). The surface epithelium showed degeneration and denudation, with loss of the microvilli. In addition there was evidence of irregularly placed nuclei, vacuolization and cellular detachment. Polymorphonucleocytes (PMN) had infiltrated the epithelium and lamina propria, and within the lamina propria blood vessels were congested and hemorrhaged. Inflammation was observed in both small intestine (duodenal) and large intestine (rectal) tissues. Inflammatory immunological responses were also evident in blood with increased PMN and leukocyte numbers and increased concentration of inflammatory mediators. Furthermore, infection resulted in strong systemic and mucosal B cell responses to *V. parahaemolyticus* lipopolysaccharide (LPS) and TDH.

The effects of *V. parahaemolyticus* on the intestinal epithelium in humans have been modeled during animal infections which has allowed characterization of the role of the TTSS in eliciting these responses. The traditional model that has been used to study the enterotoxicity of *V. parahaemolyticus* is the rabbit ileal loop model. This model has been employed to demonstrate that the bacterium is capable of more than a superficial colonization and can penetrate the lamina propria of the small intestine (Boutin et al., 1979). More recently models utilizing an orogastric route of infection have been developed. These include an infant rabbit model, an adult streptomycin-treated mouse model and a piglet model (Pineyro et al., 2010; Ritchie et al., 2012; Whitaker et al., 2012).

Ritchie et al. established a model where infant rabbits oro-gastrically inoculated with *V. parahaemolyticus* develop the severe diarrhea and inflammatory enteritis characteristic of the disease in humans (Ritchie et al., 2012). Bacteria colonized along the intestine, occurring in discrete dense clusters, presumably due to the combined actions of MSHA pili, MAM7 and other adhesins. The highest bacterial colonization and most severe pathological abnormalities were in the distal small intestine. *V. parahaemolyticus* induced severe disruption of the epithelial lining, including epithelial cell detachment and disruption of the villus structure. Inflammatory enteritis was characterized by PMN infiltration and by increased transcription of the inflammatory cytokines TNFα, IL-1β, IL-6, and IL-8. It was proposed that as *V. parahaemolyticus* recruitment of inflammatory cells occurs before widespread damage to the epithelium is evident, the inflammatory response to *V. parahaemolyticus* infection precedes, rather than occurs as a result of, extensive tissue disruption.

This model has been utilized to investigate the role of TTSS in infection. The induction of diarrhea and intestinal fluid accumulation was dependent on TTSS2 (Ritchie et al., 2012). Furthermore, the TTSS2 mutant did not elicit intestinal epithelium damage or inflammation, as neither intestinal cell sloughing nor PMN infiltration were detected. Correspondingly colonization of the intestine by *V. parahaemolyticus* was reduced in the absence of TTSS2 (Ritchie et al., 2012). TTSS1 also influenced disease progression and rates of mortality, but not to the same extent as TTSS2. The TTSS1 mutant colonized the intestine at the same level as the wild type. The incidence of diarrhea and the amount of intestinal fluid accumulation decreased in the absence of TTSS1, however, the degree of cell detachment and PMN infiltration, although reduced, was similar to the wild type.

The role of the TTSS during the infant rabbit model of infection correlates with results obtained with other animal models of *V. parahaemolyticus* intestinal infection (Hiyoshi et al., 2010). Park et al. in 2004 first demonstrated the two distinct functions of TTSS during *V. parahaemolyticus* infection (Park et al., 2004a). Using the rabbit ileal loop model they demonstrated that TTSS2 was responsible for fluid accumulation and villi destruction, with denudation of the surface epithelium. TTSS2 was also responsible for neutrophil infiltration into the lamina propria and extending up to the submucosa, resulting in congested and dilated crypts. While TTSS1 was not required for these effects *in vivo*, it was responsible for cell death in cell culture. Hiyoshi et al. also demonstrated that TTSS2 was the major contributor to *V. parahaemolyticus*-induced enterotoxicity in the adult rabbit ileal loop model, while TTSS1 had little influence (Hiyoshi et al., 2010). Orogastric inoculation of 2 day-old piglets with *V. parahaemolyticus* resulted in acute, self-limiting diarrhea and vomiting. Intestinal epithelium damage was milder than in the infant rabbit model. Fluid retention occurred in the colonic submucosa but there were no histological lesions. A TTSS2 mutant failed to produce any clinical symptoms (Pineyro et al., 2010). On the other hand a TTSS1 mutant induced symptoms to the same extent as the wild type. The TTSS1 mutant and wild type bacteria infected the gastrointestinal tract in similar numbers and both were fecally shed by the piglets, whereas bacteria were only recovered from 1 of the 3 piglets infected with the TTSS2 mutant and no fecal shedding occurred.

The results from these intestinal infection models in three animal species illustrate the TTSS2-dependency of *V. parahaemolyticus*-induced diarrhea and inflammation. TTSS2 also contributes to bacterial colonization of the intestinal tissues. TTSS1 does influence the severity of *V. parahaemolyticus* intestinal disease, however, its role is relatively minor in comparison to TTSS2.

Other animal studies have brought us further information on the roles of TTSS during infection. In a streptomycin-treated adult mouse model, prolonged colonization along the entire intestinal tract was established following orogastric infection with streptomycin-resistant *V. parahaemolyticus* (Whitaker et al., 2012). In a competition assay between wild type, TTSS1-deficient and TTSS2-deficient bacteria no difference in colonization was observed. As TTSS2 is required for intestinal colonization in other models of orogastric infection, it is unlikely that bacterial colonization is not dependent on the TTSS2 in this particular model, suggesting instead that the TTSS-mediated effects of the wild type bacteria acted in trans to allow TTSS mutant survival. This may not be unexpected as the TTSS2 effects on host cells could broadly modify the structure and function of an area of the epithelium thereby benefitting all *V. parahaemolyticus* in the vicinity, not just the bacterium that injected the effectors.

In contrast to other animal models, TTSS1 played a significant role in the lethal activity of *V. parahaemolyticus* in murine peritoneal and pulmonary infection models, while TTSS2 contributed little to the disease outcome (Hiyoshi et al., 2010; Pineyro et al., 2010). Unlike the previously described studies which are models for intestinal infection, peritoneal infection models systemic bacterial sepsis (Hiyoshi et al., 2010) and mortality in the pulmonary model is associated with pulmonary hemorrhage and systemic infection (Pineyro et al., 2010). In humans septicaemia can result when the bacteria pass across the intestinal epithelium once is has been disrupted. *V. parahaemolyticus* can also cause infection of seawater-exposed wounds. These are generally minor infections but in rare cases they can subsequently give rise to necrotizing fasciitis and septicaemia for which the mortality rate is high at 43% (Tena et al., 2010). Thus, TTSS1 is critical for *V. parahaemolyticus*-induced septicaemic lethality, and it has been proposed that the TTSS1 cytotoxic activity is the main contributor to mortality (Hiyoshi et al., 2010; Pineyro et al., 2010).

In summary the TTSS have a dramatic influence on the ability of the bacteria to colonize the intestinal tract, to induce intestinal damage leading to denudation of the microvilli from the apical surface of the enterocytes, to be enterotoxigenic and to induce inflammatory responses, such as PMN infiltration. TTSS1 influences colonization and disease outcomes to a small extent, while TTSS2 has a critical role to play in these pathogenic symptoms. The importance of TTSS2 in virulence *in vivo* corresponds to the highly frequent occurrence of the TTSS2 gene cluster in clinical isolates, while the locus is uncommon in environmental isolates.

## Specificity and variability of TTSS interactions between host cells and *V. parahaemolyticus*

Whether *V. parahaemolyticus* specifically targets enterocytes *in vivo* for TTSS deployment has not yet been directly examined, but *in vitro* studies suggest that the TTSS systems display little host cell specificity. Cytotoxicity is the most commonly used marker of TTSS1 activity, as it provides a strong and easily measured phenotype. TTSS1-mediated cytotoxicity has been detected in cell lines of several species and lineages—human colonic epithelial cells (Caco-2, HT-29, T84) (Kodama et al., 2007; Matlawska-Wasowska et al., 2010), human cervical epithelial cells (HeLa) (Park et al., 2004b), human macrophages (U937) (Zhou et al., 2009), mouse fibroblasts (3T3) (Krachler et al., 2011) and mouse monocyte/macrophages (RAW 264, J774) (Kodama et al., 2007; Hiyoshi et al., 2010). The ability of the TTSS to target cells of different mammalian species correlates with the ability of the bacterium to colonize and cause gastrointestinal infection in nematodes, pigs, rabbits and mice models, as well as in humans. TTSS2-mediated cytotoxicity can be detected in TTSS1 knockout strains. When bacteria were cultured in media that did not induce TTSS2 expression prior to co-incubation with eukaryotic cells, TTSS2-mediated cytotoxicity was observed only in human colonic epithelial cell lines (Caco-2, HCT-8) and not in HeLa, T84, J774, or non-differentiated HT-29 cells (Kodama et al., 2007), suggesting that some cell types may be more susceptible to TTSS2 cytotoxicity or may be more effective stimulators of TTSS expression, deployment or activity. In contrast the same study showed that when expressed from a multi-copy plasmid the TTSS2 cytotoxic VopT effector was translocated into each of the 6 cell types, indicating ubiquitous translocation by the TTSS. While these results may have been influenced by experimental conditions affecting TTSS and VopT expression they do suggest a broad host cell range for the *V. parahaemolyticus* TTSS which is comparable to that observed with several other mammalian gastrointestinal pathogenic bacteria, e.g., *Yersinia enterocolitica* (Boyd et al., 2000). The broad host range reflects the absence of a specific receptor for the TTSS apparatus on eukaryotic cell surfaces. Instead the host cell membrane composition determines the cellular site where the TTSS deploy (Buttner, 2012). The formation of functional TTSS translocation pores occurs preferentially in lipid raft microdomains that are rich in cholesterol and glycosphingolipids. In particular cholesterol binds the TTSS translocator proteins and is required for their assembly into an active translocation apparatus (Buttner, 2012).

While the TTSS is promiscuous, TTSS-mediated effects may be cell-specific depending on host factors and host conditions, e.g., effector target concentrations, co-factors, localization, post-translocation modifications or activation status (Mundy et al., 2007; Anderson et al., 2013). Moreover, given the intricate signaling network within eukaryotic cells, the global status of the cell will influence the behavior of the cell in response to effector-target interactions. In the case of *V. parahaemolyticus* this may result in certain cells or sections within the intestinal epithelium being more, or less, sensitive than others to the action of a particular TTSS effector.

Variation in effector gene repertoires and allelic differences in effector genes may result in variability between strains in host cell specificity and effector function. These variations may also influence the relative importance of individual effectors for the pathogenesis of a given isolate and the behaviors of the isolates themselves during infection. For example when the GtgE effector from the broad host range *Salmonella typhimurium* was introduced into the human-specific *S. Typhi*, it enabled the latter bacterium to survive and replicate within macrophages and tissues from mice, a normally non-permissive host (Spano and Galan, 2012). In *V. parahaemolyticus* two versions of TTSS2 have been identified (Okada et al., 2009). Often each co-localizes with a particular hemolysin gene. The originally identified TTSS (termed TTSS2α) co-localizes with *tdh* while the more recently identified gene cluster (termed TTSS2β) co-localizes with TDH-Related Haemolysin gene (*trh*) (Table 1). The TTSS2α phylogroup is represented by the TTSS2 in *V. parahaemolyticus* RIMD2210633 and in *V. cholerae* AM-19226, whereas the TTSS2β phylogroup is characterized by the TTSS2 in *V. parahaemolyticus* TH3996 and *V. cholerae* strains 1587 and 623-39 (Okada et al., 2009). While each TTSS2 phylogroup is important in its respective native host for enterotoxicity in the rabbit ileal loop model, there may be as yet unidentified differences in their effects on host intestinal cells.

**Table 1:** **Characterized *V. parahaemolyticus* TTSS2 effectors**.

**Effector**	**Gene**	**Biological activity**	**Effect on host cells**	***V. cholerae* AM19226 TTSS2α Homologue (% ID)^a^**	***V. cholerae* 1587 TTSS2β Homologue (% ID)**	***V. parahaemolyticus* TH3996 TTSS2β Homologue (% ID)**
VopC	*VPA1321*	Activation of Rac and Cdc42 by deamidation	Invasion of non-phagocytic cells	–^b^	VopC (48)	VopC (50)
VopT	*VPA1327*	ADP-ribosylation of Ras	Cytotoxicity	–	–	–
VopZ	*VPA1336*	Inhibition of TAK1, and downstream MAPK and NF-κB	Enterotoxicity, colonization	VopZ (50)	VopZ (25)	–
VopA/VopP	*VPA1346*	Inhibition of MAPK by acetylation of MKK	Growth inhibition in yeast	–	VopP (63)	VopP (64)
VopV	*VPA1357*	Actin binding and bundling	Enterotoxicity by fluid accumulation and blunting of villi	VopM (55)	VopM (38)	–
VopL	*VPA1370*	Actin nucleation	Formation of stress filaments, altered cell shape	VopF (62)	VopN (38)	VopL (36)

a % amino acid identity to the corresponding V. parahaemolyticus RIMD2201633 TTSS2 effector.

b Designates less than 20% amino acid identity to any protein in the strain.

## The activities of TTSS2 effectors in intestinal epithelial cells during infection

**Figure 2:**
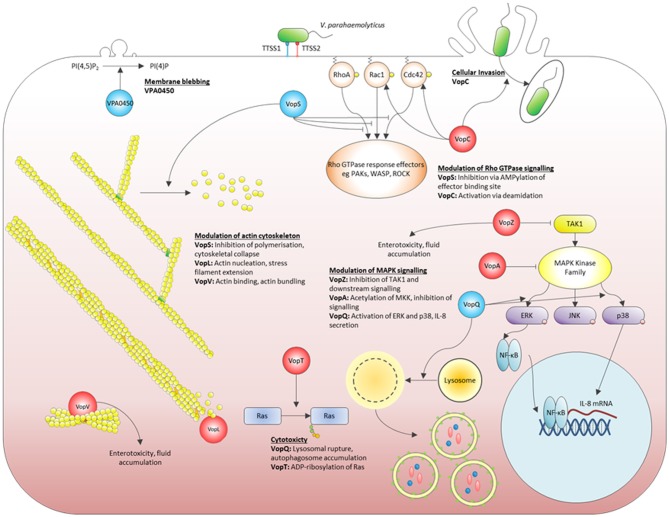
**Activity of the *V. parahaemolyticus* TTSS effectors in intestinal epithelial cells**.

Much of the research of the TTSS in *V. parahaemolyticus* had focussed originally on the TTSS1 effectors due to the unmistakable TTSS1 cytotoxic phenotype upon eukaryotic cell cultures. Now that is has been demonstrated that the induction of diarrhea, intestinal epithelial disruption and intestinal inflammation during *V. parahaemolyticus* infection is dependent on TTSS2 (Park et al., 2004b; Hiyoshi et al., 2010; Pineyro et al., 2010; Ritchie et al., 2012), recent investigations have focussed on identifying and characterizing the TTSS2 effectors. The compounding effects of TTSS1 effectors and TTSS2 effectors need to be taken into account when conducting studies to characterize the activities of these proteins during infection, as they may have additive, redundant, compensatory and/or antagonistic functions. For this reason many of the studies to elucidate the roles of the TTSS2 effectors have been performed with mutant strains of *V. parahaemolyticus* where the TTSS1 has been inactivated. This prevents TTSS-1 mediated death of eukaryotic cells at early stages of co-incubation (total elimination of living cells can occur within 4 h) and thereby allow the effects of the TTSS2 Vops on host cells to be studied in detail. Figure 2 provides a schematic representation of the diverse functionalities of the *V. parahaemolyticus* TTSS effectors which have been studied to date.

To date, six TTSS2 effectors have been identified and functionally characterized, all of which are encoded within or near the TTSS2 gene cluster (Table 1). Additional TTSS2 substrates have been identified, however, their biological activity has not yet been elucidated (Zhou et al., 2012). Two of the characterized effectors target the actin cytoskeleton directly—the actin bundling protein VopV (Hiyoshi et al., 2011) and the actin nucleator VopL (Liverman et al., 2007). Three effectors modulate the activity of eukaryotic cell signaling proteins by post-translation modification. Two of these target GTPases—VopT ADP-ribosylates the Ras GTPase (Kodama et al., 2007) and VopC deamidates Rac and Cdc42 (Zhang et al., 2012). VopA acetylates MKK proteins thereby inhibiting the Mitogen Activated Protein Kinase (MAPK) pathway (Trosky et al., 2007). While an enzymatic function has not yet been identified for the most recently characterized TTSS2 effector, VopZ, it does target the TAK1 kinase which leads into the MAPK and Nuclear Factor-κB (NF-κB) pathways (Zhou et al., 2013).

While several TTSS2 effectors have been well characterized in eukaryotic cell culture systems, fewer studies have been performed with *in vivo* models to determine the importance of individual effectors in the infection process. Like the cell culture studies of TTSS2-mediated effects on host cells, the *in vivo* studies utilized a TTSS1-deficient parental strain. VopV and VopZ were critically important for *V. parahaemolyticus*-induced enterotoxicity. In contrast single mutants of VopA, VopL, VopC, or VopT had wild type levels of fluid accumulation in the rabbit ileal loop model (Hiyoshi et al., 2011). Moreover, a triple mutant of VopA, VopL, and VopT had wild type levels of intestinal colonization and fluid accumulation in the infant rabbit model (Zhou et al., 2013).

### VopZ and VopV—required for enterotoxicity

VopV was required for *V. parahaemolyticus*-induced enterotoxicity in the rabbit ileal loop model (Hiyoshi et al., 2011). Upon infection with the VopV mutant strain fluid accumulation in the intestinal loops and signs of intestinal inflammation (epithelial loss, neutrophil infiltration in the lamina propria and submucosal area, and bleeding) were reduced to levels similar to those seen in uninfected rabbits. VopZ was necessary for fluid accumulation, cell sloughing/detachment, epithelial disruption and neutrophil infiltration in the intestine of the infant rabbit model (Zhou et al., 2013). It was also very important for bacterial colonization in this model and infected animals did not display overt signs of infection, such as diarrhea. Therefore at least two TTSS2 effectors are critical for *V. parahaemolyticus*-induced enterotoxicity.

#### VopZ

Strikingly, deletion of VopZ reduced intestinal colonization, diarrhea/fluid accumulation, and intestinal pathology to nearly the same extent as did inactivation of the entire TTSS2 (Zhou et al., 2013). VopZ inhibits activation of both the MAPK and NF-κB signaling pathways, through inhibiting activation of the TAK1 kinase (also called MAP3K7). Whether VopZ acts directly on TAK1 has not yet been elucidated. The absence of TAK1 in the intestinal epithelium results in inflammation, cell death, distorted epithelial structure and reduced barrier function indicating the importance of TAK1 for the maintenance of intestinal integrity (Kajino-Sakamoto et al., 2008). Likewise reduction of TAK1 activity by VopZ in the intestinal epithelium has a marked effect upon the integrity of this tissue and plays a critical role in the virulence of *V. parahaemolyticus*. A similar protein occurs in *V. cholerae* (Table 1) where it may play a related role in pathogenesis. The degree of sequence similarity differs considerably for the two *V. cholerae* TTSS2 phylogroups. As with the effectors VopV and VopL, the protein sequence of *V. parahaemolyticus* RIMD2210633 (phylogroup TTSS2α) VopZ is more similar to the protein in *V. cholerae* AM19226 (which belongs to the same phylogroup), than to *V. cholerae* 1589 which belongs to phylogroup TTSS2β (Table 1). Interestingly *V. parahaemolyticus* TH3996 of phylogroup TTSS2β does not appear to possess a VopZ homologue. The NF-κB and MAPK signaling pathways regulate immune responses to bacterial infection and other danger signals. However, while PMN infiltration *in vivo* was reduced with the VopZ mutant, cytokine levels were comparable to the wild type (Zhou et al., 2013). Moreover, the biological activity of VopZ that determines its importance for colonization and PMN infiltration seems to be separable from the TAK1-inhibition activity that is responsible for fluid accumulation, cell detachment and epithelial damage (Zhou et al., 2013). This suggests that *V. parahaemolyticus* induces inflammation by alternative cell signaling pathways.

#### VopV

VopV was the first TTSS2 effector found to be required for *V. parahaemolyticus* enterotoxicity (Hiyoshi et al., 2011). Despite having a profound effect on enterotoxicity *in vivo*, VopV is not involved in TTSS2-dependent cytotoxic activity to Caco-2 cells, indicating that the cytotoxic activity of TTSS2 does not correlate with its enterotoxic activity. VopV's mode of action is distinct from that of VopZ, as it targets filamentous actin (F-actin) rather than a cell signaling protein. The actin cytoskeleton is a major determinant of the shape of a eukaryotic cell, and it is highly dynamic with continuous assembly and disassembly of actin filaments, thus allowing the cell to rapidly change morphology in response to external cues. Manipulation of actin dynamics and actin structure by pathogens can lead to dramatic changes in cell structure and tissue structure, with consequent abnormal cell and tissue behaviors (Haglund and Welch, 2011).

VopV disrupted actin dynamics in transfected cells by directly binding F-actin and bundling it into concentrated foci (Hiyoshi et al., 2011). The effector possesses two F-actin binding domains—a C-terminal domain and a long repeat (LR) domain—and the LR domain alone has the ability to accumulate thick bundles of parallel actin filaments. Mutant bacteria lacking VopV did not cause fluid accumulation or blunting of rabbit ileal villi upon infection (Hiyoshi et al., 2011). Infection with *V. parahaemolyticus* expressing VopV possessing just one of either of the actin binding domains resulted in restoration of fluid accumulation demonstrating that the actin binding function of VopV (rather than the actin bundling) was responsible for enterotoxicity. VopV is similar to VopM of *V. cholerae* which plays a similar role in inducing enterotoxicity (Hiyoshi et al., 2011).

The mechanism by which F-actin binding of VopV leads to enterotoxicity is not yet understood. The enterotoxicity may be due directly to the disruption of the actin cytoskeleton leading to epithelial disruption and fluid loss. In addition, or alternatively, the effect may be indirect through cytoskeletal disruption bringing about changes in the activation status of signal transduction pathways that lead to fluid loss. Recent studies have highlighted the role of actin interacting/modifying proteins in the process of wound healing in intestinal tissues and in regulation of intestinal cell death (Wang et al., 2012; Rankin et al., 2013; Ubelmann et al., 2013). Wound healing involves cell remodeling, which includes cell migration and microvillus depolarization—features that are observed during *V. parahaemolyticus* infection. Microvillus depolarization occurs through the action of the actin binding and bundling protein villin and villin also plays a key role in controlling cell death through regulating actin dynamics. It would be interesting to investigate if the ability of VopV to bind F-actin would enable or impede the involvement of F-actin in wound healing in intestinal tissue. Further research in this area will prove invaluable in determining the mechanism by which *V. parahaemolyticus* induces secretory diarrhea in infected individuals.

### VopL, VopA, VopT and VopC—powerful modulators of host cell activity

Although VopL, VopA, VopT, or VopC are not required for *V. parahaemolyticus*-induced diarrhea or for colonization in rabbit models of infection, their biochemical functions and their effects on epithelial cell lines strongly supports the hypothesis that they are important for manipulating host cell behavior during infection.

#### VopL

VopL is the second actin targeting TTSS2 effector, in particular modifying actin assembly. The assembly of actin fibers is a complex process that involves an initial nucleation step requiring three or more actin monomers that then serve as a priming site for further polymerization of the actin filament (Haglund and Welch, 2011). Eukaryotic actin-nucleating proteins accelerate the initial nucleation step. VopL mimics these to potently and directly facilitate the assembly of actin (Liverman et al., 2007). VopL possesses three proline-rich motifs and three WASP homology 2 (WH2) domains, both of which are found in eukaryotic actin nucleators (Liverman et al., 2007). Transfection of VopL into epithelial cells induced stress fiber formation and VopL co-localized with the actin fibers. Like its eukaryotic counterparts VopL independently binds and nucleates globular actin *in vitro* (Liverman et al., 2007). Indeed it induced more rapid extension of actin fibers, and was active at lower concentrations, than the eukaryotic nucleators SPIRE and Arp2/3, indicating that VopL is a more potent activator of actin assembly than host proteins, thereby enabling *V. parahaemolyticus* to override actin homeostasis in infected cells (Liverman et al., 2007).

All three WH2 domains, as well as the C-terminal domain of VopL (VCD), are required for actin nucleation (Namgoong et al., 2011; Yu et al., 2011). Recent structural determinations of VopL and VopL domains in isolation or bound to actin have provided information on the molecular basis for the function of actin nucleators (Rebowski et al., 2010; Namgoong et al., 2011; Yu et al., 2011; Zahm et al., 2013). Isolated WH2 domain repeats and isolated VCDs can mediate actin recruitment but are thought to be intrinsically weak nucleators. Therefore, the domains act synergistically to catalyze actin nucleation. During nucleation the VCD contributes directly to the low-affinity recruitment of actin subunits. The VCD also promotes VopL dimerization and thereby brings together six WH2 domains, each of which binds an actin subunit with high affinity so that the VopL dimer stabilizes an actin nucleus to which further subunits can be added. After nucleation VopL quickly dissociates and is not involved in the processive elongation of the actin filament. In this way VopL promotes extremely fast cycles of filament nucleation.

Similar, but not identical, actin nucleating proteins exist in *V. cholerae*—VopF and VopN (Tam et al., 2007, 2010). Amino acid sequence analysis reveals two groups of VopL proteins in *V. parahaemolyticus* isolates. The first group is highly similar to the VopL of *V. parahaemolyticus* RIMD2210633 (99–100% identity) and belongs to phylogroup TTSS2α. This group shares 57% identity with the *V. cholerae* VopF of the TTSS2α phylogroup. Modulation of actin dynamics by the *V. cholerae* VopF is important for the colonization of the small intestine during *in vivo* infections of mice (Tam et al., 2007). In an epithelial cell line VopF caused the formation of actin-rich filopodial projections from the periphery of the cells, with VopF located at the tip of these protrusions. Consistent with these features, a model has been proposed whereby the VopF WH2 domains uncap the terminal ends of the actin filament to allow elongation (Tam et al., 2007; Pernier et al., 2013). The second group of *V. parahaemolyticus* VopL proteins has identity to the *V. parahaemolyticus* RIMD2210633 VopL protein in the region of 38%, corresponding mainly to the C-terminal 350 amino acids, and belongs to phylogroup TTSS2β. It shares a large degree of identity (87–95%) with the VopN of *V. cholerae* TTSS2β phylogroup. Unlike VopF, the *V. cholerae* VopN co-localized to stress fibers and bundled actin cables at the dorsal cell surface (Tam et al., 2010). By the use of VopF-VopN fusions it was shown that the NH_2_-terminal amino acids determine the effector's subcellular localization. Like the *V. parahaemolyticus* VopL, the VopN mutant was not defective in *V. cholerae* colonization in a suckling mouse competition assay, nor was it required for fluid accumulation in a rabbit ileal loop model. Further investigation to establish the significance of VopL during infection will yield new insights into the importance of actin remodeling during *V. parahaemolyticus* pathogenesis.

#### VopA

VopA is a member of the YopJ protein family, which comprises TTSS effectors of several animal and plant pathogens, including the YopJ effector of *Yersinia pestis* (Makino et al., 2003). YopJ is an acetyltransferase which binds to and acetylates the critical serine and threonine residues of the MAPK kinase (MKK) superfamily, thereby preventing the subsequent activation of MAPK and NF-κB (Orth et al., 1999). The YopJ family can promote cell death by blocking these signaling pathways (Trosky et al., 2004, 2007). VopA inhibits the three main MAPK cell signaling pathways—ERK, p38 and JNK—however, it does not affect the activation of NF-κB (Trosky et al., 2004, 2007). Expression of VopA in yeast resulted in inhibition of growth, and its expression in Jurkat T cells caused cell death, both features reminiscent of the *Yersinia* YopJ. VopA inhibits MKKs in two ways. It prevents their activation and also inhibits the kinase function of already activated MKKs. Focusing specifically on MKK6, the kinase which phosphorylates the ERK MAPK, it was shown that VopA acetylates four residues in this protein—Ser-207, Lys-210, and Thr-211 in the activation loop and Lys-172 in the catalytic loop (Trosky et al., 2007). Acetylation of Ser-207 and Thr-211 prevents phosphorylation of these amino acids and thereby prevents activation of the kinase. Acetylation of Lys-172 prevents ATP binding to the MKK, and it is then unable to modify its substrates. While VopA does not play a role in *V. parahaemolyticus* colonization or diarrhea, its importance for modulating innate immune responses to infection via inhibition of the MAPK merits further study.

#### VopT

The TTSS2 effectors VopT and VopC target the low molecular weight G proteins which are involved in the regulation of cell proliferation, vesicular trafficking and cytoskeletal homeostasis. VopT is an ADP-ribosyl transferase that modifies Ras GTPase by the transfer of ADP-ribose from NAD+ to the substrate protein (Kodama et al., 2007). Ras was the only one of the 14 small GTPases tested that was modified by VopT, showing a high degree of substrate specificity. VopT is similar to the ExoS and ExoT TTSS ADP-ribosyltransferases of *Pseudomonas aeruginosa*, though each effector has its own GTPase substrate specificity (Barbieri and Sun, 2004). ExoS and ExoT require binding of the eukaryotic co-factor 14-3-3 to their Fas domain for activity (Coburn et al., 1991). Similarly the Fas domain of YopT was required for its enzymatic activity. Expression of VopT resulted in inhibition of growth in yeast and Δ*vopT* mutants were found to have decreased TTSS2-mediated cytotoxicity. The precise mechanism by which Ras modification leads to cell death has yet to be determined (Kodama et al., 2007).

#### VopC

VopC shows similarity to the catalytic domain of cytotoxic necrotizing factor (CNF) toxins. CNF toxins induce changes in the actin cytoskeleton and facilitate invasion of pathogens into host cells (Aktories and Barbieri, 2005) through their deamidase/transglutaminase activity toward Rho family GTPases (Flatau et al., 1997). The activation of Rac1 and Cdc42 via transamidation by VopC was dependent on Cys-220 within the effector's catalytic domain and resulted in modifications to the actin cytoskeleton of infected HeLa cells (Zhang et al., 2012). Zhang et al. identified that TTSS2, and specifically VopC, was required for the invasion of HeLa cells by *V. parahaemolyticus* (Zhang et al., 2012). These results indicate that VopC functions to facilitate invasion in a similar manner to the *Salmonella* TTSS effector protein SopE by modulating actin dynamics through the activation of Rac1 and Cdc42 (Friebel et al., 2001). VopC homologues are present in TTSS2β phylotype *V. cholerae* strains and have a similar role in promoting *V. cholerae* invasion into HeLa cells. The fate of the intracellular bacteria and their significance during infection is an open question.

## The activities of TTSS1 effectors in cultured intestinal epithelial cells

While TTSS2 has been clearly linked to enterotoxicity in *in vivo* mammalian infection models, a role for TTSS1 in intestinal infections has not yet been established. This is surprising given the biochemical activities of the TTSS1 effectors detected in eukaryotic cell culture lines. Three TTSS1 effectors—VopQ, VopS, and VPA0450—have been functionally characterized to date (Table 2). TTSS1 is possessed by all *V. parahaemolyticus* strains, regardless of their pathogenic potential. As such it has been proposed that TTSS1 may be mainly responsible for *V. parahaemolyticus* interactions with hosts, such as amoeba or other marine organisms, that it encounters in its native habitat (Zhang and Orth, 2013). Systemic infections of *V. parahaemolyticus*, though rare, do occur and TTSS1 is required for lethality in animal models representing systemic infection (Hiyoshi et al., 2010; Pineyro et al., 2010). Though TTSS1 seems not to be required for intestinal infection in animal models, it does influence the severity of the disease and may therefore be important for the final outcome of the infection and long-term effects on human health (Ritchie et al., 2012).

**Table 2:** **Characterized *V. parahaemolyticus* TTSS1 effectors and their role in pathogenesis**.

**Effector**	**Gene**	**Biological activity**	**Effect on host cells**
VopQ/VepA	*VP1680*	Binds V-ATPase and forms pores in lysosomal membranes	Lysosomal deacidification, autophagy, cell lysis, activation of MAPK, secretion of IL-8
VopS	*VP1686*	Inhibition of Rho GTPases by AMPylation	Rounding of host cells, invasion of phagocytes
VPA0450	*VPA0450*	Phosphatidylinositol phosphatase	Membrane blebbing and destabilization in HeLa cells

### VopQ

VopQ is the most extensively studied of all the *Vibrio* TTSS effectors. VopQ was identified initially in 2004 as the predominant protein secreted by the *V. parahaemolyticus* TTSS1 (Park et al., 2004b), and over the past decade its effect on host cells has received much attention from researchers.

The principle cytotoxic effector of *V. parahaemolyticus* is VopQ, encoded by *VP1680* (Ono et al., 2006). Several cell death mechanisms have been proposed for the route of VopQ cytotoxicity, the most recent of which is disruption of lysosomal homeostasis brought about by interaction of VopQ with the V-ATPase in the lysosomal membrane (Matsuda et al., 2012; Sreelatha et al., 2013). A yeast genome-wide screen identified subunit c of V-ATPase as essential for the growth inhibition of yeast by VopQ and in HeLa cells knockdown of V-ATPase subunit c (ATP6V0C) decreased VopQ cytotoxicity (Matsuda et al., 2012). V-ATPases are located in the membranes of intracellular organelles where they act as proton pumps to acidify the vesicles (Mindell, 2012). VopQ inhibited V-ATPase proton translocation (Sreelatha et al., 2013), thereby unbalancing proton gradients and causing deacidification of organelles. Microinjection of recombinant VopQ into HeLa cells was sufficient to cause lysosomal deacidification within 20 min (Sreelatha et al., 2013) and extended exposure ultimately led to lysosomal rupture and subsequently cell lysis (Matsuda et al., 2012). Recently this model has been refined by Sreelatha et al. (2013) who showed that VopQ forms small pores in lysosomal membranes. These pores are 18Å gated outward rectifying channels which allow passage of particles of 350–3000 Da (Sreelatha et al., 2013). Electrostatic interactions between VopQ and the negatively charged lipids in the lysosomal membrane favor formation of a stable VopQ channel. The association of VopQ with lipids at physiological pH is restricted to membranes containing the V_o_ domain of the V-ATPase. This creates a protective mechanism against promiscuous VopQ pore-forming activity in the bacterium and the host.

VopQ-mediated lysis of infected cells is associated with VopQ-dependent induction of autophagy (Burdette et al., 2009a). Autophagy is the process by which cells degrade and recycle cytoplasmic contents. This occurs first by encapsulation of the targeted material in a distinctive double bilayer membrane vesicle (the autophagic vesicle), and then delivery of the vesicle to the lysosome for degradation. Microinjection of recombinant VopQ into host cells resulted in the accumulation of autophagic vesicles, illustrating that VopQ alone is sufficient for *V. parahaemolyticus*-induced autophagy, (Burdette et al., 2009a). Burdette et al. speculated that the induction of autophagy by *V. parahaemolyticus* may provide a means of sequestration of cellular material in order to facilitate rapid *in vivo* growth (Burdette et al., 2009b). Although autophagy is well-known to promote cell survival in response to various stimuli, autophagy also plays a role as an executor of cell death. However, evidence suggests that VopQ-mediated cell lysis does not occur as a consequence of autophagy. In HeLa cells, inhibiting autophagy with short interfering RNAs (siRNAs) targeting the autophagy mediator ATG5 did not affect TTSS1 cytotoxicity (Matsuda et al., 2012). Moreover, Δ*atg5* and Δ*atg8* yeast strains, both of which are deficient in the autophagic process, were not resistant to VopQ toxicity (Matsuda et al., 2012). Instead it has been proposed that the autophagy ensuing upon *V. parahaemolyticus* infection is a protective reaction of the host cell in response to the VopQ-mediated release of lysosomal contents into the cell cytosol (Matsuda et al., 2012). An alternative proposal is that VopQ prevents degradation of the autophagic vesicles rather than stimulating their generation. This is due to lysosome-dependent turnover of autophagic vesicles being inhibited by VopQ-mediated lysosomal deacidification (Sreelatha et al., 2013). The result is the accumulation of toxic, structurally disruptive and energy-draining structures and cellular components. Ultimately, cells in which damaged proteins or organelles accumulate die because of bioenergetic and metabolic dysfunction (Jegga et al., 2011).

TTSS1 also plays a role in the induction of inflammatory chemokines. VopQ stimulates the secretion of the chemokine Interleukin 8 (IL-8) by differentiated Caco-2 cells in response to *V. parahaemolyticus*, via the activation of MAPK (Matlawska-Wasowska et al., 2010; Shimohata et al., 2011). IL-8 is secreted basolaterally by epithelial cells to attract neutrophils to the site of infection and it is a key player in the initiation of an inflammatory response. The MAPK activation also contributes to VopQ-mediated cell lysis. The MAPK activation could be due to the cellular stress occurring as a consequence of lysosomal rupture (Matsuda et al., 2012). On the other hand bacterial pore-forming toxins can directly regulate MAPK pathways, and perhaps VopQ can act in this way (Porta et al., 2011). Alternatively VopQ could induce IL-8 secretion through its function as a lysosomal membrane pore-forming toxin which rapidly disturbs cytosolic ion concentrations, leading to MAPK activation (Sreelatha et al., 2013).

VopQ thus has a number of dramatic effects on host cells. Its importance in systemic models of infection, where TTSS1 is critical, as well as its importance for innate immune responses during intestinal infection, merit further study.

### VopS

Yarbrough et al. identified a novel protein post-translational modification when characterizing VopS (Yarbrough et al., 2009). The Rho GTPases, RhoA, Rac1, and Cdc42, were modified by the addition of the adenosine monophosphate portion of ATP to a conserved threonine residue (AMPylation). The modified GTPases were no longer able to interact with downstream substrates due to steric hindrance by the covalently linked AMP moiety, leading ultimately to collapse of the actin cytoskeleton (Casselli et al., 2008). As such, the characteristic cell rounding associated with *V. parahaemolyticus* infection was attributed to VopS. VopS has also been associated with immune suppression. During recognition of invading pathogens by the innate immune system, a multi-protein complex called the inflammasome is assembled (Kim and Jo, 2013), which triggers pro-inflammatory responses in host cells. While the TDH hemolysins in combination with the TTSS induce inflammasome activation upon *V. parahaemolyticus* infection of macrophages (Higa et al., 2013), VopQ-mediated autophagy and VopS-mediated GTPase inhibition suppress inflammosome activity. The C terminus of VopS contains a Fic domain which is responsible for AMPylation activity (Luong et al., 2010). Fic domains are evolutionarily conserved from prokaryotes to eukaryotes and VopS is now the archetypal representative of this family (Woolery et al., 2010).

### VPA0450

VPA0450 causes rapid cell rounding and lysis of HeLa cells (Broberg et al., 2010). Hydrolysis of the D5 phosphate from the inositol ring of the membrane-associated lipid phosphoinositol (4,5) bisphosphate (PI[4,5]P_2_) by VPA0450 induced the formation of protruding blebs in the host cell membrane (Broberg et al., 2010). PI(4,5)P2 plays a critical role in the regulation of cell signaling events at the plasma membrane (Krauss and Haucke, 2007). As such, Broberg et al. hypothesized that VPA0450 may play a complementary role with VopS and VopQ in host cell rounding and lysis by destabilizing plasma membrane-cytoskeleton dynamics at late stages of infection (Broberg et al., 2010). VPA0450 has not yet been given a “Vop” designation.

## Conclusion

The recent publications characterizing *V. parahaemolyticus* virulence factors provide a clearer picture of the bacterium's pathogenicity (Figure 2). Upon infection of the human host *V. parahaemolyticus* utilizes a number of factors—MSHA pili, MAM7, cHA, T6SS, CPS, GbpA—to attach to the epithelial cells lining the intestinal tract so that colonization can be initiated. Of these adhesins MAM7 and the MSHA pili interact with fibronectin, phosphatidic acid and specific glycans to establish cell-contact between the bacterium and the eukaryotic cell so that a fully functional translocation system can be built for the delivery of TTSS effectors into the host cell cytosol. The TTSS2 effectors VopV and VopZ bundle actin filaments and inhibit TAK1 kinase, respectively, to bring about disruption of the intestinal epithelial layer and cause diarrhea. Damage to the epithelial layer features cell sloughing and microvilli denudation, and associated with this damage is an inflammatory response, characterized by the influx of PMNs to the site of infection. The inflammatory response is stimulated at least in part through the activation of MAPK by VopQ, which would seem to override the inhibition of the MAPK pathway by the TTSS2 VopA and VopZ. Cell sloughing may be a consequence of the capacity of the TTSS1 effectors VPA0450 and VopS to promote abberant cell morphologies and cell rounding, through their effects on phosphoinositides and the actin cytoskeleton. The actin cytoskeleton is also modified by the TTSS2 VopL actin nucleator producing stress fibers. Alongside this induction of intestinal dysfunction, a proportion of cells in the epithelium will be invaded by *V. parahaemolyticus* through the targeting of Rho GTPases by TTSS2 VopC. Other cells in the intestine will be killed primarily by the action of TTSS1-secreted VopQ on lysosome deacidification, and abetted by the inhibition of the Ras GTPase by TTSS2-secreted VopT. All in all, the barrage of TTSS effectors represents a powerful weaponry by which *V. parahaemolyticus* can elicit disease.

Research on the biological activities, function and structure of the adhesins and TTSS effectors is rapidly advancing our understanding of the molecular mechanisms of *V. parahaemolyticus* virulence. This exciting new knowledge provides us with a picture of the interactions that occur between a pathogenic bacterium and intestinal epithelial cells and opens new avenues for exploration in the future.

### Conflict of interest statement

The authors declare that the research was conducted in the absence of any commercial or financial relationships that could be construed as a potential conflict of interest.
